# Application and Experimental Validation of a Multibody Model with Weakly Coupled Lateral and Vertical Dynamics to a Scaled Railway Vehicle

**DOI:** 10.3390/s20133700

**Published:** 2020-07-01

**Authors:** Pedro Urda, Sergio Muñoz, Javier F. Aceituno, José L. Escalona

**Affiliations:** 1 Department of Mechanical and Manufacturing Engineering, University of Seville, 41092 Sevilla, Spain; escalona@us.es; 2 Department of Materials and Transportation Engineering, University of Seville, 41092 Sevilla, Spain; sergiomunoz@us.es; 3 Department of Mechanical and Mining Engineering, University of Jaén, 23071 Jaén, Spain; jaceitun@ujaen.es

**Keywords:** multibody model experimental validation, scaled railway vehicle, scaled track, instrumented wheelset

## Abstract

In this paper, a multibody dynamic model of a railway vehicle that assumes that vertical and lateral dynamics are weakly coupled, has been experimentally validated using an instrumented scaled vehicle running on a 5-inch-wide experimental track. The proposed linearised model treats the vertical and lateral dynamics of the multibody system almost independently, being coupled exclusively by the suspension forces. Several experiments have been carried out at the scaled railroad facilities at the University of Seville in order to test and validate the simulation model under different working conditions. The scaled vehicle used in the experiments is a bogie instrumented with various sensors that register the accelerations and angular velocities of the vehicle, its forward velocity, its position along the track, and the wheel–rail contact forces in the front wheelset. The obtained results demonstrate how the proposed computational model correctly reproduces the dynamics of the real mechanical system in an efficient computational manner.

## 1. Introduction

The presence of computer simulations applied to the dynamic analysis of railroad vehicles and infrastructures has increased in the railways industry over the last decade. This is due to the appearance of new and sophisticated multibody models that allow very realistic simulations of almost any kind of railway vehicle. In fact, the most recent updates of European and international standards for vehicle approval, such as the EN-14363 [1] or the UIC-518 [2], allow the use of computational simulations during the certification process of new railroad vehicles. This is a very interesting point from an economic perspective, because manufacturers can reduce the number of expensive field tests with real vehicles running on test tracks. Naturally, this is possible whenever the accuracy of the model used for the certification has been proven in advance. As explained by Polach et al. in [3], the robustness and precision of any multibody simulation model, as simple as it is, can only be ensured once it has been experimentally validated with real measurements unless an analytical solution exists, which is rarely the case of general railway dynamics.

Since their first appearance in 1916, railway simulation models have been steadily growing and evolving. Nowadays, there is commercial software that is specifically developed for precise rolling stock dynamic simulations such as GENSYS, MEDYNA, NUCARS or VAMPIRE, and general purpose multibody software like ADAMS or SIMPACK with extensions for railway simulations [4]. Despite this, some researchers are committed to the development of their own simulation codes. There are a large number of references in the scientific literature concerning this subject and addressing it from different perspectives. One example is the work of Escalona et al. [5,6,7] which analyses the dynamics of the vehicle while interacting with the track from a theoretical point of view. Other works [8] combine theory and experiments trying to achieve a more precise estimation of wheel-rail contact forces from the inertial response of the vehicle. Railway simulation models are normally founded on the most classical theories [9,10,11] and complex mathematical approaches. However, despite their sophistication and theoretical accuracy, their performance compared to the real mechanical system is in most cases yet to be proven. Some railway simulation models found in the literature are validated with field tests. In the work of Lombaert et al. [12], the railway induced vibrations on the track-soil are experimentally measured on a commercial line between Brussels and Köln and compared with simulation results. A similar experimental validation of induced vibration estimated with a metro line simulation model can be found in [13].

The experimental validation of a railroad computational model requires experiments to be carried out using real vehicles running on commercial tracks [14]. This is something that is normally difficult to achieve due to the fact that operating companies pay a high price to conduct such experiments. For safety reasons, test vehicles cannot be in commercial operation with passengers during an experimental campaign, and that can result in substantial economic losses for the company. An intermediate solution is the use of full scale roller-rigs that simulate the vehicle while interacting with the track [15,16]. However, roller-rigs are rare and expensive equipment not available to every research institution or railway manufacturing company. In this regard, many researchers use scaled vehicles running on scaled track or roller-rigs as a way to analyse the dynamic response of the vehicles. One example of this is the work of Kim et al. [17] where a 1:5 scaled vehicle running on a scaled track is used to analyse the performance of a steering control system for Independent Rotated Wheels (IRW) vehicles. The same scaled facility is also used in [18] to develop an intelligent traction and brake control system that improves the performance of the vehicle when negotiating very sharp radius curves similar to those found in many commercial metro lines. Another interesting thesis is presented in [19] by Bosso et al. where a 1:4 scaled prototype on a roller-rig is used to analyse the dynamics of narrow gauge railway vehicles. The experimental results confirm the theoretical predictions using SIMPACK. In [20] Iwnicki and Wickens present a simple dynamic model developed in *MATLAB*^®^ whose results are validated using a 1:5 scaled vehicle. This vehicle is tested on an experimental roller-rig that allows the simulation of track irregularities by means of hydraulic actuators. Although the use of a scaled systems is not fully representative of the real mechanical system, it has the advantage of being an scenario where the test conditions (track geometry, irregularities, wheel-rail profiles, vehicle’s parameters, etc.) are known in advance, something that is not always possible with a real vehicle and track.

In addition to its precision when reproducing the dynamic performance of the real physical system, any railway multibody simulation model is also intended to be computationally efficient. The most sophisticated and commonly used commercial simulation software in the railroad industry and research, such as *SIMPACK* or *ADAMS-Rail*, despite their accuracy, are far from having Real-Time (RT) simulation capabilities. This represents a clear drawback if they want to be implemented on a vehicle’s onboard computer for the online running-safety monitoring of the vehicle, for instance. In this regard, having a simple but yet precise railroad simulation model that is able to run in RT represents a clear competitive advantage [21]. The increase in the service speed and volume of rail transport over the last few decades has required not only an improvement in the comfort of the vehicles, but also in the mechanics and safety of both the vehicles and the rails. Under these circumstances, the life cycle of many railway components has grown shorter causing a significant increase in maintenance costs. Every railway operating company is interested in on-board systems for the continuous monitoring of their vehicles and tracks for the detection of derailment at its earliest stage (detecting the magnitude of the vertical (V) and lateral (L) wheel-rail contact forces that can be used to calculate the value of derailment coefficients, like Nadal formula, to evaluate the risk of wheel climbing) [22,23,24], estimation of wheel and rail profiles [25], detection of track defects [26], or the measurement of track irregularities. Any of these methods must be developed on the basis of a robust, accurate, and RT capable simulation model.

In this paper, the experimental validation of a railroad multibody simulation model is presented. The mentioned model has been fully developed by the Department of Mechanical Engineering at the University of Seville [7]. It assumes that vertical and lateral dynamics are weakly coupled through the suspension forces. In a previous phase of this work, the performance of this linearised model was compared with simulation results drawn from a full non-linear 3D model showing promising results. The main advantage of the reduced model is its higher computational efficiency when compared to a fully non-linear 3D model. The ultimate goal of this work is to demonstrate the accuracy and robustness of the simplified model in a real scenario. For that purpose, a set of experiments have been carried out using an instrumented 1:10 scaled vehicle [27] running on an experimental scaled track at different forward velocities. The vehicle is instrumented with two inertial sensors that register the acceleration and angular velocity of the leading wheelset and the bogie frame respectively, two distance lasers that measure the deflection experienced by the primary suspension, two position encoders that register the forward velocity and travelled distance, and a dynamometric wheelset for the measurement of wheel-rail contact forces. The experimental results have been compared with the numerical results drawn from the simulation model showing a good accordance between experiments and simulations.

The manuscript is organised as follows. Section 2 briefly describes the proposed simplified computational multibody model. Section 3 presents the experimental scaled track and instrumented vehicle used in the experiments. Section 4 shows a comparison between simulations and experiments carried out in the space and frequency domain. Section 5 summarizes the conclusions obtained in this work.

## 2. Dynamic Modelling

The dynamic model used in this work was previously presented by the authors in [7]. A brief description of the model is given next. The dynamic model is a general formulation for railway vehicles running on tracks with arbitrary geometry with irregularities. Due to the fact that the model has been developed with future industrial applications in mind, the number of required parameters has been minimised. As a result, the proposed model is general, complete and computationally efficient due to the following features:

1. It is based on the use of track-relative unconstrained coordinates for each vehicle body. Generalised coordinates are separated into vertical coordinates and lateral coordinates. Bodies are also split into *wheelsets* and *non-wheelset* bodies.
2. Kinematic linearisation (small-angles assumption) and dynamic linearisation of inertia and suspension generalised forces is performed.
3. It considers weakly coupled local vertical and lateral dynamics of the vehicle through the suspension forces. This is, vertical dynamics is solved to obtain the suspension forces first. Then, these forces are used to solve the lateral dynamic. Note that, the coupling comes from the suspension forces. Relative-longitudinal dynamic is ignored.
4. Wheel-rail contact interaction is based on the equivalent conicity concept, the *knife-edge contact* assumption [6,28], and Polach’s rolling contact theory. In addition, flange contact and two-point contact scenarios are considered using an elastic penetration-based contact approach using a Hunt-Crossley model [29] that considers an elastic lateral force based on the flange and rail surfaces penetration and penetration rate. More details about flange contact can be found in [7].
5. Equations of motion are obtained using symbolic computations. The computation of generalised forces is optimised using symbolic computation techniques.

**Figure 1:**
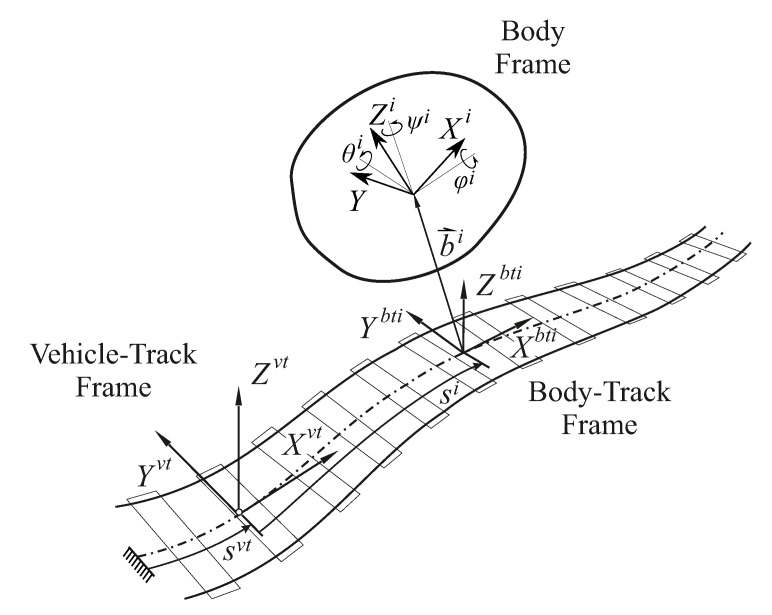
Frames of reference and kinematic description of arbitrary vehicle body.

In this multibody model, the motion of the bodies is described with respect to an intermediate frame that is neither inertial nor body fixed, which is called Track Frame (TF). The TF has its origin at the track centreline and its *X* axis is parallel to its tangent. The TF used in this work is not a Frenet frame of the track centreline since the selected *Y* axis is not in the direction of the centre of curvature but it connects the left and right rail centrelines. The track centreline is defined assuming an ideal reference geometry with absence of irregularities. In the model formulation, several frames are used to obtain the equations of motion (see Figure 1). Firstly, the Vehicle-Track Frame (VTF) is a moving TF that advances along the guide-way with approximately the same forward velocity as the vehicle. Additionally, each body *i* of the vehicle has an associated Body-Track Frame (BT*i*F) that is a moving TF that accompanies the motion of the body along the track centreline. The track coordinates describe the position and orientation of the Body *i* Frame (B*i*F) with respect to the BT*i*F. These coordinates are composed of the position vector $b^{i}=\left[x^{i},y^{i},z^{i}\right]$ and the orientation components $\Phi =\left[\varphi^{i},\theta^{i},\psi^{i}\right]$. Notice that, in the formulation of the equations of motion described next, the *i* superscript must be replaced by *wi* when the body is a wheelset, or by *nwi* when the body is a non-wheelset.

The equations of motion are given by:

(1) $$M_{V}^{nw}\;{\ddot{q}}_{V}^{nw}\;+C_{V}^{s,nw}{\dot{q}}_{V}^{nw}+K_{V}^{s,nw}q_{V}^{nw}=Q_{V}^{ForIn}-C_{V}^{s,w}{\dot{q}}_{V}^{w}-K_{V}^{s,w}q_{V}^{w}+Q_{V}^{grav}+Q_{V0}^{s}$$

(2) $$M_{L}\;{\ddot{q}}_{L}\;+\left[C_{L}^{s}+C_{L}^{c}\right]{\dot{q}}_{L}+\left[K_{L}^{s}+K_{L}^{c}\right]q_{L}=Q_{L}^{ForIn}+Q_{L0}^{s}+Q_{L0}^{c}+Q_{L}^{grav}$$

where Equation (1) includes the equations of motion for the vertical dynamics, $q_{V}^{nw}$ being the generalised coordinates associated with the vertical dynamics of the non-wheelset bodies and $q_{V}^{w}$ are the generalised wheelset coordinates associated with the vertical dynamics, given by the track’s vertical geometry. Unlike the vertical coordinates of the non-wheelset bodies $q_{V}^{nw}$, the set $q_{V}^{w}$ is not considered as generalised coordinates since its value is determined through the *knife-edge contact* constraints. Both sets of coordinates are described as follows:

(3) $$q_{V}^{nwi}={\left[\begin{array}{ccc} z^{nwi} & \varphi^{nwi} & \theta^{nwi} \end{array}\right]}^{T}$$

(4) $$q_{V}^{wi}={\left[\begin{array}{cc} z^{wi} & \varphi^{wi} \end{array}\right]}^{T}$$

where *i* stands for body *i*. It should be noted that, the rolling angle $\theta^{wi}$ is assumed to vary such that $\dot{\theta }={\dot{s}}^{vt}/r_{0}$, where ${\dot{s}}^{vt}$ is the forward velocity and $r_{0}$ is the rolling radius of the wheels when the wheelset is centred on the track.

In Equation (1), $M_{V}^{nw}$, $C_{V}^{nw}$ and $K_{V}^{nw}$ respectively represent the constant mass, suspension damping and suspension stiffness matrices associated with the vertical dynamics; $C_{V}^{s,w}$ and $K_{V}^{s,w}$ respectively represent the constant suspension damping and suspension stiffness matrices associated with the wheelset vertical coordinates; $Q_{V}^{ForIn}$ is the vector of generalised inertia forces due to the forward motion in the vertical direction; $Q_{V}^{grav}$ is the generalised gravity force vector; and $Q_{V0}^{s}$ contains the constant terms that appear in the generalised suspension forces. Equation (2) includes the equations of motion for the lateral dynamics, $q_{L}$ being the lateral generalised coordinates of the vehicle bodies that are described as follows:

(5) $$q_{L}^{i}={\left[\begin{array}{cc} q_{L}^{nwi} & q_{L}^{wi} \end{array}\right]}^{T}={\left[\begin{array}{cccc} y^{nwi} & \psi^{nwi} & y^{wi} & \psi^{wi} \end{array}\right]}^{T}$$

Terms $M_{L}$, $C_{L}^{s}$ and $K_{L}^{s}$ in Equation (2) respectively represent the constant mass, suspension damping and suspension stiffness matrices associated with the lateral dynamics, $C_{L}^{c}$ and $K_{L}^{c}$ are damping and suspension matrices associated with the contact forces acting on the wheelset in the lateral direction; $Q_{L}^{ForIn}$ represents the vector of generalised inertia forces due to the forward motion; the vectors $Q_{L0}^{s}$ and $Q_{L0}^{c}$ contain the terms that appear in the generalised suspension forces and in the generalised contact forces, respectively, when the lateral coordinates and velocities are zero; and $Q_{L}^{grav}$ is the vector of generalised gravity forces in the lateral direction.

The equations of motion of the vertical and lateral dynamics of the vehicle (Equations (1) and (2)) can be solved separately although they are coupled through the suspension forces and the wheel-rail constraints. It is important to note that the arc-length longitudinal coordinate of each body of the vehicle, $s^{i}$, has not been included in the set of generalised coordinates, $q_{V}$ and $q_{L}$. The reason for this is that in the proposed model that neglects the relative-longitudinal dynamics of the bodies, the value of $s^{i}$ is naturally obtained by prescribing the longitudinal motion of the vehicle, for example, using a forward velocity profile. Furthermore, the model accounts for the influence of the longitudinal motion in the lateral dynamics of the vehicle using the vector of generalised inertial forces, $Q_{L}^{ForIn}$, that depends on $ds^{i}/dt$ and $d^{2}s^{i}/dt^{2}$.

**Figure 2:**
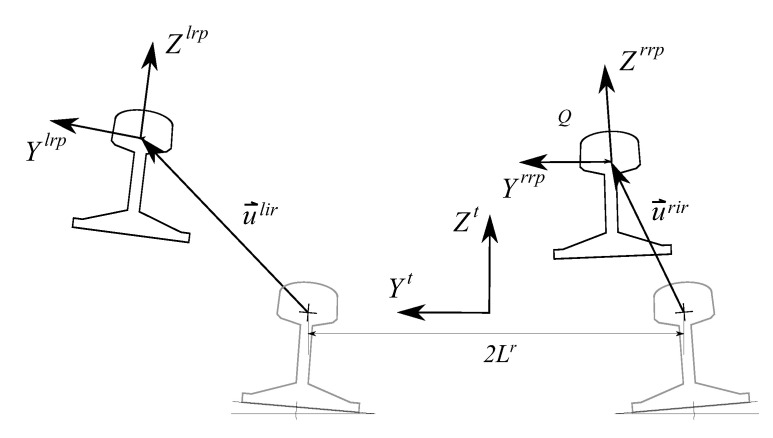
Definition of track irregularities.

As previously mentioned, the present dynamic model can include track irregularities, which are deviations from the ideal track geometry that can cause unwanted vehicle dynamic responses leading to poor ride quality or, possibly, to safety problems. These irregularities can be divided in two types, lateral and vertical, and are usually described using four variables: track gauge variation ($\xi_{g}$) and lateral alignment ($\xi_{a}$) for lateral irregularities, and cross-level ($\xi_{cl}$) and vertical profile ($\xi_{vp}$) for vertical irregularities. These variables are defined as follows:

(6) $$\begin{array}{c} \xi_{g}=\left(u_{y}^{lir}-u_{y}^{rir}\right),\;\xi_{a}=\left(u_{y}^{lir}+u_{y}^{rir}\right)/2\\ \xi_{cl}=\left(u_{z}^{lir}-u_{z}^{rir}\right),\;\xi_{vp}=\left(u_{z}^{lir}+u_{z}^{rir}\right)/2 \end{array}$$

where $u_{y}^{lir}$, $u_{y}^{rir}$, $u_{z}^{lir}$ and $u_{z}^{rir}$ represent the lateral (‘*y*’) and vertical (‘*z*’) deviations of the left (‘lir’) and right (‘rir’) rail cross-sections from their ideal positions, see Figure 2.

## 3. Experimental Validation

As previously stated, the main goal of this paper is to experimentally validate the railways multibody model proposed in Section 2. As explained by Polach et al. in [30], the most precise way to validate any railroad dynamic model requires experimenting with the real mechanical system. Taking into account that it is not always possible to use a real railway vehicle for experimental purposes, in this work, a 1:10 scaled railway vehicle running on an experimental scaled track has been used as an alternative to validate the aforementioned dynamic model. It is important to note that due to the large scale reduction with respect to a real railroad vehicle, it is not the intention of this paper to extend the dynamic results obtained with the scaled system to full scaled units. In this section, the main features of the experimental scaled track and the instrumented vehicle are presented.

### 3.1. Experimental Scaled Track

**Figure 3:**
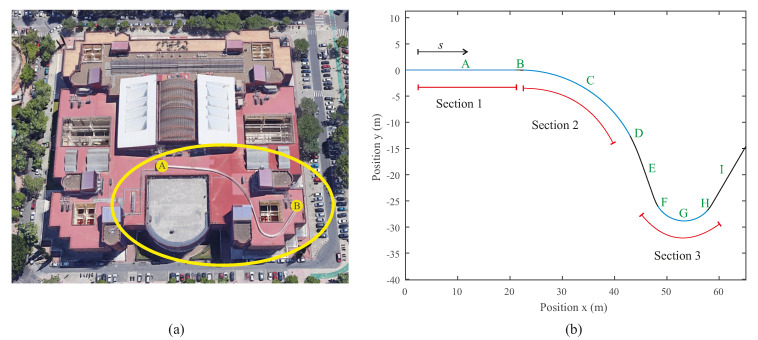
Separate views of the scaled track: aerial photograph (**a**) and sketch of the track centre line (**b**).

The experiments have been carried out on a 90 m long, 5-inch-wide scaled track installed on the rooftop of the School of Engineering at the University of Seville. Figure 3a shows an aerial view of the building where the experimental track can be observed. The scaled track geometry intends to emulate a real one including a combination of tangent and constant radius sections linked with variable curvature sections as normally found on a real track. Table 1 summarizes the different sections of the Track Centre Line (TCL). Figure 3b shows the horizontal projection of the scaled track highlighting the three regions of greatest interest for the later dynamic analysis. In these three sections, it can be assumed that the vehicle reaches a permanent regime.

**Figure 4:**
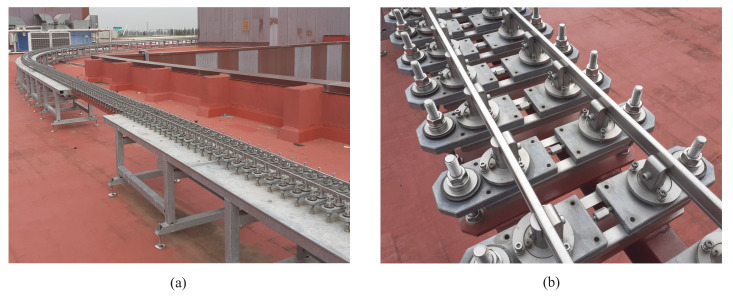
Scaled track assembly (**a**) and mechanical sleepers (**b**).

The track has been installed over a set of metallic benches distributed along the rooftop of the building (see Figure 4a). The rails are held using 900 mechanisms that emulate a conventional track sleeper. However, these mechanisms have the property that they allow the manual insertion of arbitrary track irregularities on the track. Adjusting the different nuts and bolts of the mechanical sleeper (see Figure 4b), the track gauge, cant angle and relative height between both rails can be modified. In order to compensate the centrifugal force experienced by the vehicle when it negotiates both curves, two constant cant angles of 0.5 and 2.5 degrees respectively have been set in both constant curvature sections assuming an average forward velocity of 1.5 m/s.

**Figure 5:**
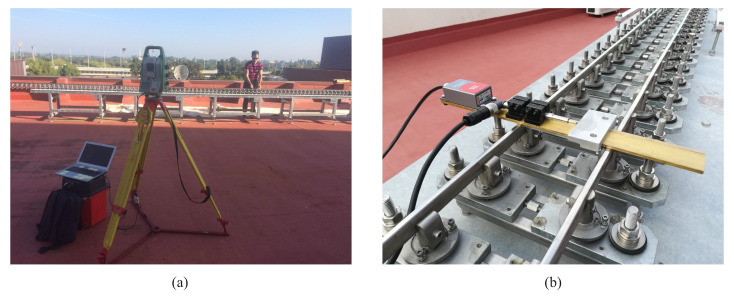
Author measuring the TCL with the robotic total station (**a**). Track gauge and cant angle measuring device (**b**).

**Figure 6:**
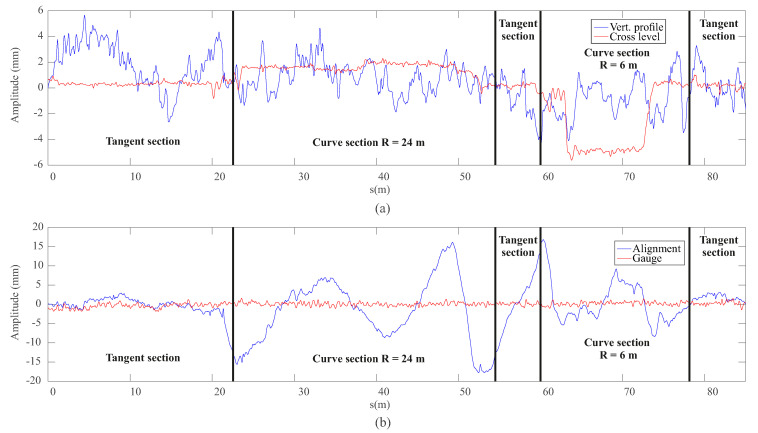
Vertical (**a**) and lateral (**b**) track irregularities.

One of the main advantages of experimenting with a scaled systems lies in the fact that test conditions can be precisely determined in advance. This is an important point in order to reduce model uncertainties during the validation process. The experimental scaled track centre line has been carefully measured using a high-precision, robotic total station *Leica Nova MS50* and a prism (see Figure 5a). Then, using a Linear Variable Differential Transformer (LVDT) and an inclinometer (see Figure 5b), the track gauge and cant angle have been measured along the track. Finally, the difference between the measured geometry and the real geometry constitute the track irregularities. Figure 6 shows the obtained vertical profile, cross level, alignment, and gauge irregularities which are those normally used in the industry. As it can be observed in Figure 6, the values of the global irregularities (lateral alignment and vertical profile), are considerably higher than those of relative irregularities (track gauge variation and cross-level). Solid vertical lines in Figure 6 define different track section according to Table 1. In order to avoid making the figures too crowded with many vertical lines, transition sections are not marked in Figure 6 and next. Transition sections are considered to be included in the curve sections highlighted in the figures. The measured track geometry and its irregularities are then used in the multibody model as inputs.

### 3.2. Instrumented Scaled Vehicle

**Figure 7:**
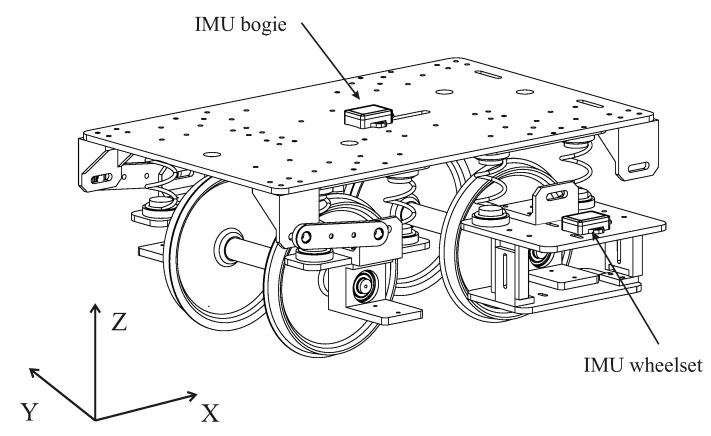
CAD of the instrumented scaled vehicle.

**Figure 8:**
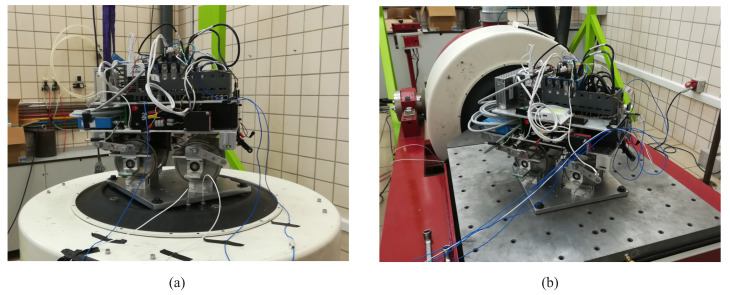
(**a**) Experiment in the vertical direction. (**b**) Experiment in the lateral direction.

The 1:10 scaled railway vehicle used for the multibody model experimental validation has been designed and manufactured by the Department of Mechanical Engineering at the University of Seville. The scaling strategy followed during the design process can be found in [31]. Figure 7 shows the CAD design of the aforementioned vehicle. The vehicle is intended to reproduce a conventional bogie [9,32], including two wheelsets and a bogie frame connected through the primary suspension. There are also two traction rods that connect the rear wheelset with the bogie frame. Their purpose is to reduce the longitudinal oscillations created by the transmission on the rear axle. The vehicle parameters have been carefully measured in the laboratory. The different bodies the vehicle includes have been weighed separately. The total mass of the entire vehicle including all its instruments is 18.4 kg. Inertia parameters have been determined through CAD-software. The stiffness and damping parameters of the suspension elements have been measured experimentally using a test machine and experimental modal analysis. In addition the vehicle has been tested on a vibration platform (see Figure 8) in vertical and lateral directions in order to obtain the different vibration modes of the instrumented vehicle. The vehicle has a total length of 440 mm and a height of 350 mm, including its instrumentation. The wheels have a nominal radius of 63.5 mm.

**Figure 9:**
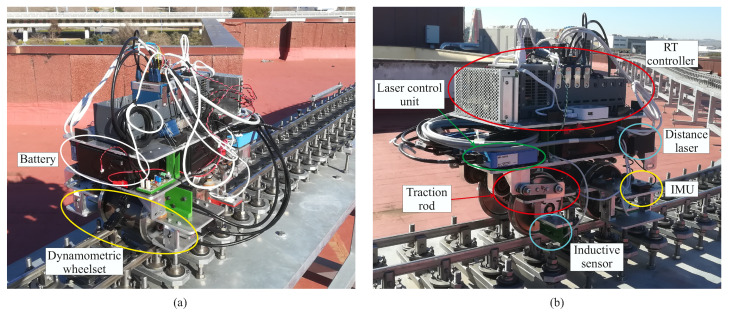
Front end (**a**) and back end (**b**) of the instrumented vehicle.

The vehicle has been instrumented with several sensors whose measurements are later used in the vehicle’s dynamic analysis. Figure 9 shows the final assembly of the scaled vehicle on the track. As it can be observed, its final look differs from the geometry shown in Figure 7 due to all the sensors and instruments installed on it. The vehicle has two Inertial Measurement Units (IMUs). The first one is installed on the front-right bearing box and the second one in the geometric centre of the bogie frame (see Figure 7 and Figure 9). The IMUs register the acceleration and angular velocity experienced by both vehicle’s bodies where they are installed. There are also two precision lasers that measure the deflection experienced by the front wheelset primary suspension. Two encoders register the instantaneous velocity and angular position of both wheelsets. The vehicle also has a couple of inductive sensors that detect the position of several beacons distributed every two meters along the track. The positions of such beacons were measured using the total station and the prism during the measurement process of the track. The measurements of the encoders along with the inductive sensors are inputs of the odometer system that precisely locate the vehicle on the track. Every time the vehicle passes a beacon, the instantaneous position registered by the encoder is compared with the real position of the beacon at the track and updated if necessary. Finally, a dynamometric wheelset (front axle) instrumented with strain gauges and high-precision distance lasers, as shown in [27], measures the instantaneous applied lateral force. The vehicle is powered by a 24 W DC motor and a set of lead-acid batteries. A chain transmission connects the output gear of the motor and the rear axle. A NI CompactRIO-9035 of National Instruments, an industrial controller with Real Time (RT) capabilities, controls the vehicle’s forward velocity using the measurement of the precision encoder installed in the front axle and a regular PI control algorithm. This RT controller is also in charge of acquiring the sensors’ measurements and storing the acquired data. The acquisition frequency has been set to 500 Hz, the maximum frequency allowed by the IMUs.

## 4. Simulations to Experiments Comparison

**Figure 10:**
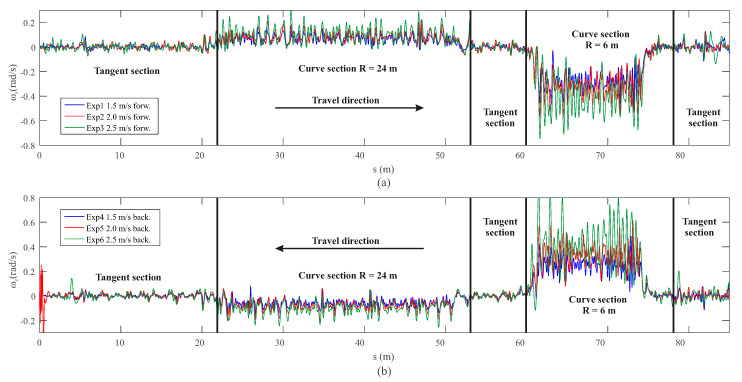
Measured yaw angular velocity of the leading axle in the forward (**a**) and backwards experiments (**b**).

In this section, the experimental validation and performance analysis of the proposed dynamic multibody model is presented. An experimental campaign of six tests has been carried out in the scaled track facilities at the University of Seville. The trials account for different conditions: three forward velocity levels (1.5 m/s, 2.0 m/s and 2.5 m/s) and both directions of travel (forward and backward). Hereinafter, experiments where the vehicle moves from point A to point B in Figure 3a are named as experiments in the forward direction of the track. The opposite direction, from point B to point A, is considered backwards movement of the vehicle on the track. In order to not overload the manuscript with dozens of graphs, this work only shows the results obtained from one of the six experiments carried out. The conclusions drawn from this particular case can be extended to the other five experiments. As an example, Figure 10 shows the measured yaw angular velocity in the experimental campaign. As it can be observed, all the experiments yielded similar results.

**Figure 11:**
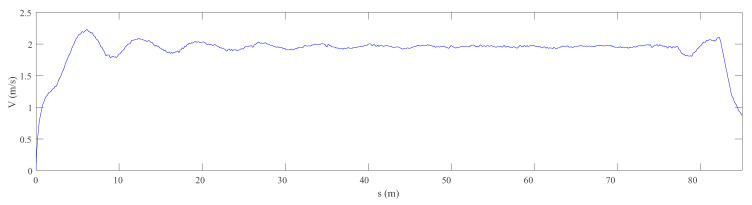
Forward velocity profile.

In the experiment chosen for the analysis, the vehicle moves in the forward direction of the track at an approximately constant velocity *V* = 2.0 m/s. The maximum distance travelled was 85 m. It must be noted that, even though the velocity set-point is a constant value, the actual forward velocity slightly changes during experiment due to the starting and braking stages as well as the oscillations due to the motor PI control as observed in Figure 11.

The computational model under analysis requires the vehicle’s parameters (such as masses, inertias, suspension properties, etc), the ideal track geometry and its irregularities, and the travelled distance and forward velocity of the vehicle during the experiments (drawn from the odometer) as inputs.

The experimental validation presented in this section entails a comparison between the simulated accelerations, angular velocities and applied forces on the instrumented wheel drawn from the computational model and the equivalent experimental results measured on the track with the two IMUs and the dynamometric wheelset installed on the vehicle. In addition, a frequency analysis that compares simulations and experiments has been also carried out in the three sections of the track where the vehicle reaches a stationary state. These three track sections are depicted in Figure 3. Section 1 (tangent) is located between *s* = 5–20 m, Section 2 (constant radius R = 24 m) between *s* = 23–47 m, and Section 3 (constant radius R = 6 m) between *s* = 63–73 m.

**Figure 12:**
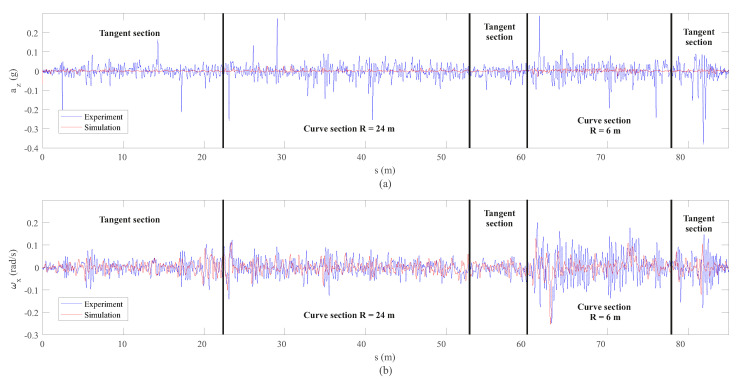
Vertical acceleration (**a**) and roll angular velocity (**b**) data from inertial sensors on the wheelset.

**Figure 13:**
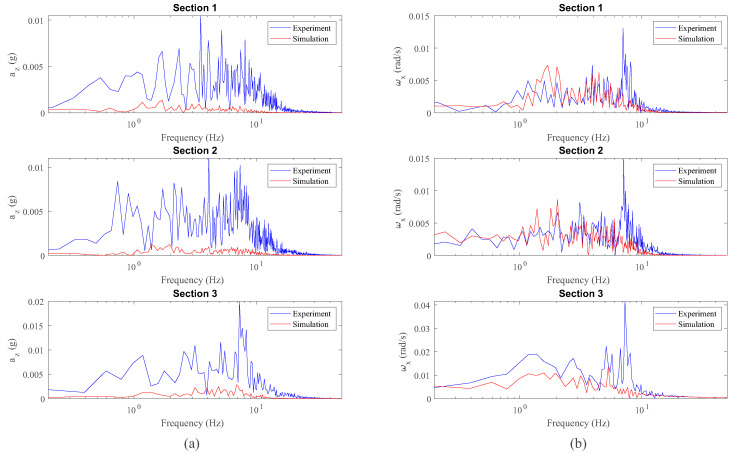
Frequency analysis of vertical acceleration (**a**) and roll angular velocity (**b**) data from inertial sensors on the wheelset.

Vertical and lateral dynamic responses have been analysed separately. Figure 12 shows a comparison between the vertical acceleration $a_{z}$ and roll angular velocity $\omega_{x}$ experienced by the leading wheelset during the experiment represented as a function of the distance travelled *s*. Then, Figure 13 shows the comparison between experiments and simulations in the frequency domain.

As it can be observed in Figure 12a, the simulated vertical acceleration, $a_{z}$, is considerably smaller than the measured component. This phenomenon can also be observed in the frequency domain (see Figure 13) in the three sections of the track under analysis. This event can be explained as follows: the irregularities have been experimentally measured on the track every 50 mm, meaning that the irregularities with wave lengths smaller than 100 mm can not be detected by the simulation model. These short wave length irregularities normally lead to significant vertical acceleration on the rolling stock and, consequently, on the inertial sensor mounted on the wheelset as depicted in Figure 12a. The induced vertical acceleration can be amplified not only in the frequency corresponding to the excitation but in the whole frequency range [33]. This fact has been confronted by the authors, through simulations with the inclusion of a short wavelength corrugation. In addition, there are more factors that influence in the induced vertical accelerations, such as track flexibility (the model considers the track rigid), wheel out-of-roundness, wheel flexibility, effect of the transmission and misalignments produced in the vehicle assembly (i.e., suspension attachments, bearings, etc.).

**Figure 14:**
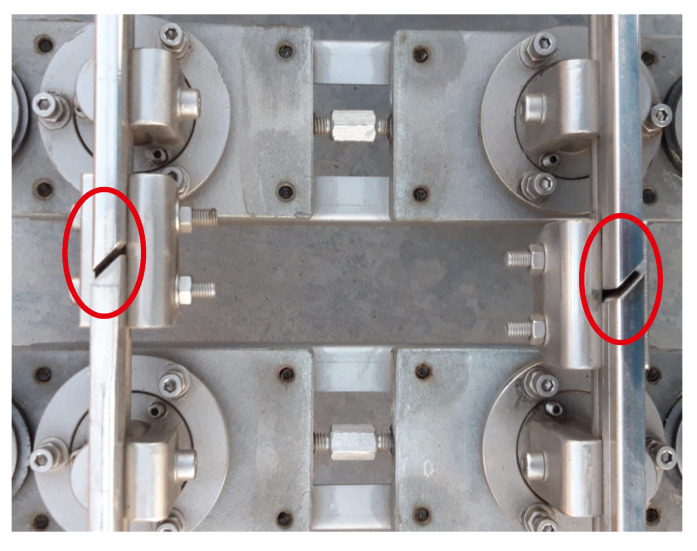
Largest gap between two consecutive rail sections of the track.

Some acceleration peaks distributed through the experiment can also be observed in Figure 12a. Those peaks are due to the small gap between each two consecutive rail sections. Those gaps were intentionally introduced during the track assembly process in order to compensate for the effects of temperature (see Figure 14). It must be noted that the track is located outdoors and exposed to very high temperatures during the summer months in Seville (Spain). Although the positions of these gaps are perfectly identified along the track, they are not included in the dynamic model, so they cannot be identified in the simulation results. In order to avoid uncertainties due to the effects of temperature, both the track measurements and experiments were carried out under similar ambient temperature conditions.

Regarding the measured roll angular velocity (see Figure 12b), it can be said that the experimental results confirm the simulated data. This is also confirmed in the frequency domain analysis depicted in Figure 13b where a good match is observed in the whole spectrum, excluding a peak in the experiments at a frequency of 7.1 Hz which is not detected by the model. This frequency of 7.1 Hz is observed in both experimental signals in Figure 13: vertical acceleration, $a_{z}$, and roll angular velocity, $\omega_{x}$. This peak, not detected by the proposed model, is mainly related to a vibration mode associated with the pitch rotation and the longitudinal displacement of the different bodies, and subsequently it is reflected in $a_{z}$ and $\omega_{x}$. Since the relative-longitudinal dynamics are neglected in the model, the relative-longitudinal motion between the bodies is not considered and, consequently, the boundary conditions differ from reality. This fact makes the proposed model unable to capture the frequency peak of 7.1 Hz attributed to the pitch rotation and the longitudinal displacement of the bodies. This explanation has been corroborated by the use of a full 3D coupled dynamic model previously proposed by the authors [5] which has been able to capture this vibration mode. An extended comparison between the simplified model proposed in this manuscript and the mentioned full-3D multibody model is presented by the authors in [7].

**Figure 15:**
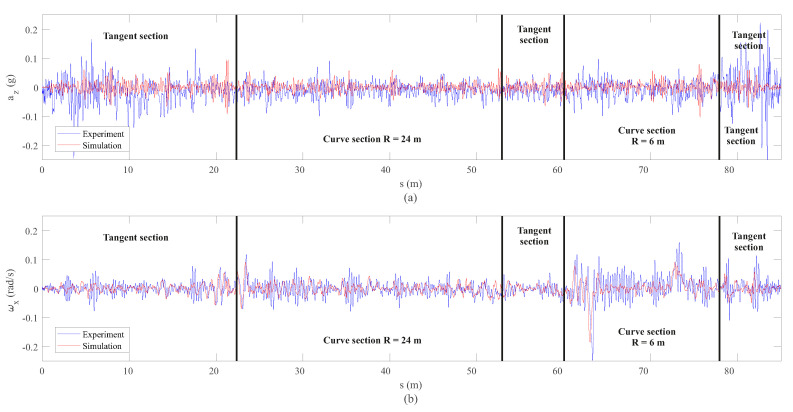
Vertical acceleration (**a**) and roll angular velocity (**b**) data from inertial sensors on the bogie frame.

**Figure 16:**
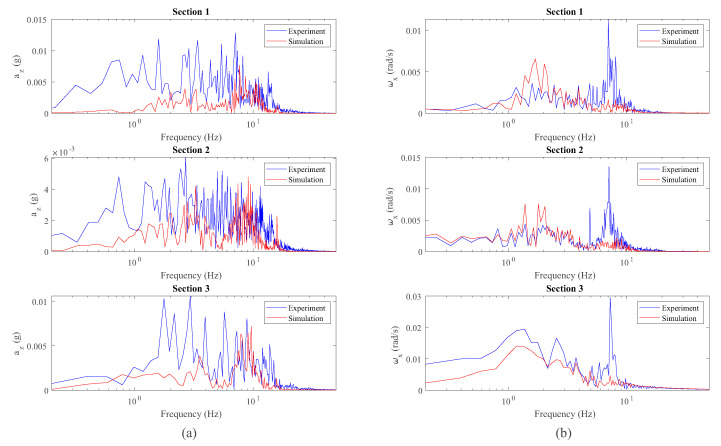
Frequency analysis of vertical acceleration (**a**) and roll angular velocity (**b**) data from inertial sensors on the bogie frame.

An equivalent analysis has been carried out with the measurement of IMU installed in the bogie frame. Figure 15 shows the comparison between the measured vertical acceleration, $a_{z}$, and roll angular velocity, $\omega_{x}$, experienced by the bogie frame and the equivalent simulated data. A frequency domain analysis has also been considered in this case, and its results are shown in Figure 16.

The conclusions drawn from the bogie frame’s vertical dynamics are similar to those obtained in the wheelset dynamic analysis. In the case of the vertical acceleration in the bogie frame, $a_{z}$, the results obtained in simulations are lower than the experimental results as can be observed in the space domain, Figure 15, and in the frequency domain, Figure 16. These results are related to the underestimation of $a_{z}$ in the wheelsets, since the vertical acceleration in both bodies, wheelset and bogie frame, is closely related to the primary suspension. Regarding the roll angular velocity in the bogie frame, $\omega_{x}$, good results are obtained in simulations as can be observed in the space domain, Figure 15, and in the frequency domain, Figure 16. Again, a peak at a frequency of 7.1 Hz is observed in both experimental signals in Figure 16 ($a_{z}$ and $\omega_{x}$) which is not detected by the model due to the reasons previously explained.

**Figure 17:**
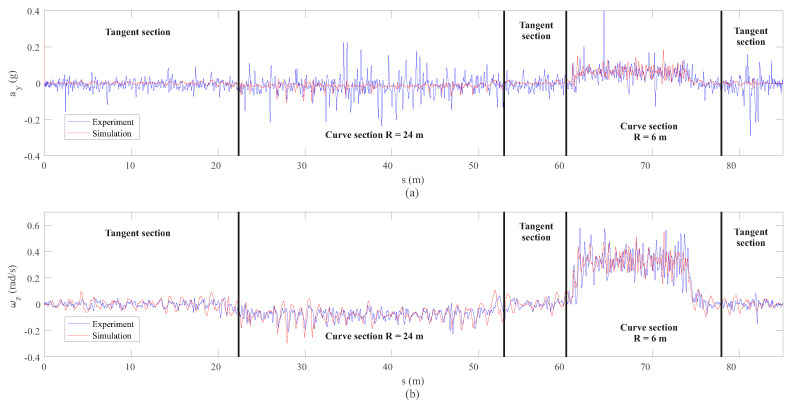
Lateral acceleration (**a**) and yaw angular velocity (**b**) data from inertial sensors on the wheelset.

**Figure 18:**
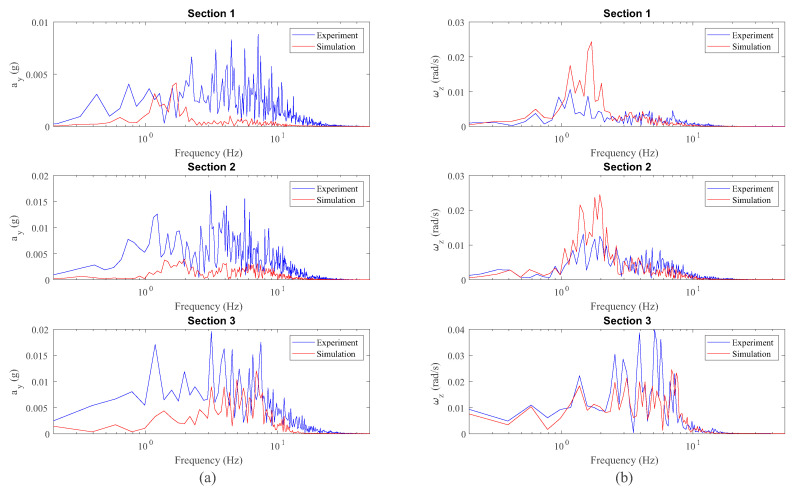
Frequency analysis of lateral acceleration (**a**) and yaw angular velocity (**b**) data from inertial sensors on the wheelset.

After analysing the results of the vertical dynamics, the experimental validation of the lateral dynamics is presented next. The measurements of both IMUs installed on the front wheelset and bogie frame respectively have been compared with the simulation results drawn from the computational model. Figure 17 shows the comparison in the space domain between the lateral acceleration $a_{y}$ and the yaw angular velocity $\omega_{z}$, corresponding to the IMU placed on the front wheelset. On the other hand, Figure 18 shows the same comparison but in the frequency domain for each section of interest.

Several conclusions can be drawn from the figures above. As expected, it can be seen in Figure 17 how both sensors, the accelerometer and the gyroscope, are able to detect both curves: the open curve (*s* = 23–47 m) and the sharp curve (*s* = 63–73 m). In view of the results in the space domain shown in Figure 17, a quite good agreement is achieved between experiments and simulation, especially in the case of the yaw angular velocity, $\omega_{z}$. These results can be corroborated with the frequency analysis in Figure 18. Another interesting result is the good agreement in the detection by both the simulation model and the experiments, of the hunting phenomenon, an unstable oscillatory movement with both variables, lateral displacement, *y*, and yaw angle, ψ, 90° out of phase. This oscillatory hunting movement has a frequency of 1.7 Hz as can be extracted from both measurements ($a_{y}$ and $\omega_{z}$) in Figure 18. This phenomenon fits quite well with what is predicted by using the well-known Klingel’s formula [34].

**Figure 19:**
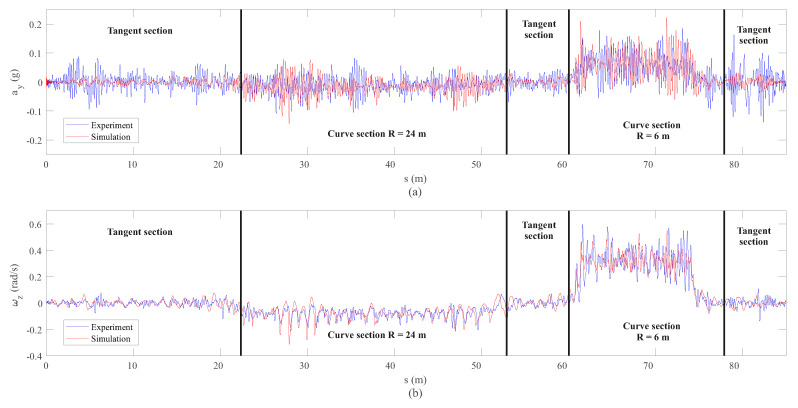
Lateral acceleration (**a**) and yaw angular velocity (**b**) data from inertial sensors on the bogie frame.

**Figure 20:**
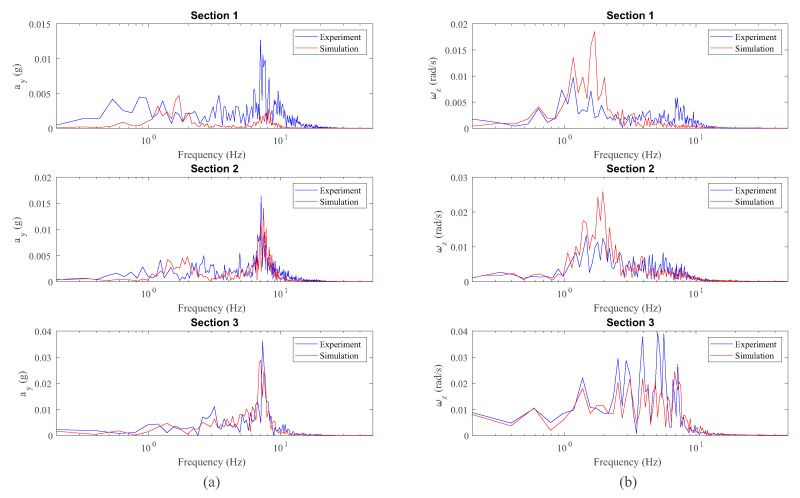
Frequency analysis of lateral acceleration (**a**) and yaw angular velocity (**b**) data from inertial sensors on the bogie frame.

Next, the lateral acceleration, $a_{y}$, and the yaw angular velocity, $\omega_{z}$, corresponding to the IMU on the bogie are presented in Figure 19 in the length domain and in Figure 20 in the frequency domain.

Results very similar to those obtained in the dynamic analysis of the wheelset are encountered. Quite good agreement is achieved for the angular velocity vectors experimental measurements and simulations as observed in the length domain (Figure 19) and in the frequency domain (Figure 20). Again, the hunting frequency is properly detected by both experimental measurements and simulations as can be seen in Figure 20. Additionally, a peak in frequency around 7.5 Hz corresponding to the lateral acceleration is observed in Figure 20. This peak in frequency corresponds to the relatively high lateral stiffness of the primary suspension between the wheelset and the bogie frame.

**Figure 21:**
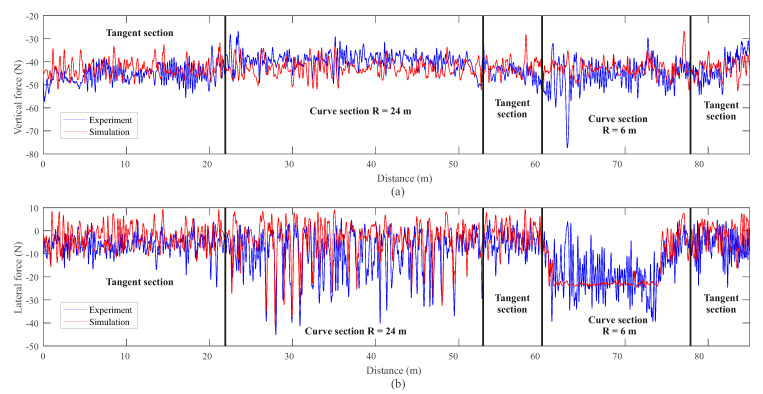
Instrumented wheel vertical (**a**) and lateral (**b**) applied forces.

The final elements used to validate the model’s performance are the measurements of the dynamometric wheelset. This axel has its front-left wheel instrumented with several force transducers, as explained in Section 3.2, that register the instantaneous vertical and lateral forces applied on it. The obtained results of vertical and lateral applied forces versus the distance travelled are shown in Figure 21. It can be observed how the vertical load remains at around a quarter of the total weight of the vehicle. Both signals show an acceptable accordance. Figure 21b depicts the applied lateral force on the instrumented wheel. In this case, an even more robust agreement between experiments and simulations is obtained. In the large radius curve, it can be observed how the model closely matches the instantaneous impacts between the wheel and the rails produced by the intermittent lateral displacement of the leading wheelset while negotiating the curve section. In the sharp radius curve, a continuous flange contact is estimated by the simulation model. In this latter case, the experimental measurement shows larger fluctuation compared to the model estimation, although the mean values of both signals coincide.

**Figure 22:**
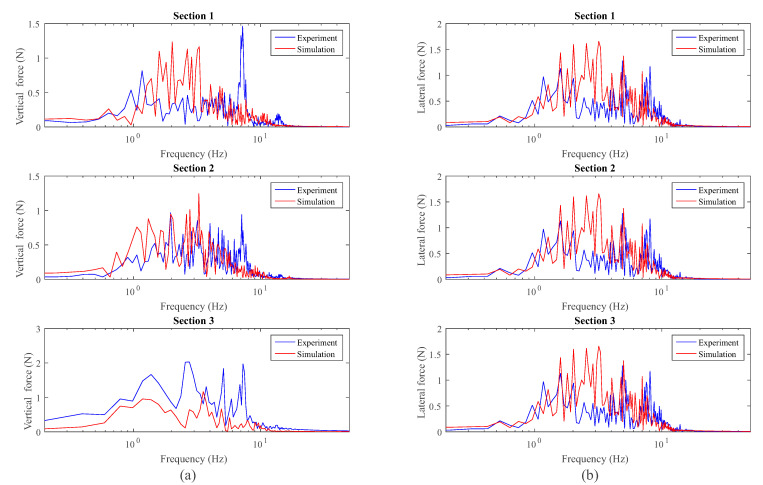
Frequency analysis of the applied vertical (**a**) and lateral (**b**) forces.

Finally, Figure 22 shows the frequency analysis of the measured forces compared with the simulation results. The analysis has been again divided in the three sections of the track where the vehicle reaches the quasi-stationary state. In this figure, a peak at a frequency of 7.1 Hz is observed in the experimental vertical force which is not detected by the model due to the reasons previously explained. As it was observed before with the inertial measurements, the applied forces also show a good accordance with the numerical data when they are represented in the frequency domain.

## 5. Summary and Conclusions

In this paper, the authors present the experimental validation of a railways multibody simulation model in order to demonstrate the model’s capabilities. The proposed model assumes that vertical and lateral dynamics are weakly coupled through the suspension forces. That assumption allows the independent solution of the dynamics equations in both directions. Vertical dynamics is solved first and suspension forces obtained. These forces are then used as inputs in the lateral dynamics equations. This methodology results in an improvement in the computational efficiency of the model. As shown in [7] the reduced formulations is up to 3 times faster than a full 3D multibody model formulation. The experimental validation has been carried out using a scaled railway vehicle instrumented with several sensors which moves on an open experimental scaled track built on the rooftop of the School of Engineering at the University of Seville. The experiments have been carried out in both directions of the track using different forward velocities. The registered velocity profile during the experiments along with the track geometry, its irregularities, and the vehicle’s parameters, are used as inputs for the computational simulation model. The proposed experimental validation is based on the direct comparison and frequency analysis between simulations and sensors’ measurements. The analysed signals are accelerations, angular velocities, and applied forces on the instrumented wheel.

In view of the obtained results, it can be said that the computational model satisfactorily reproduces the scaled railway vehicle’s dynamics. It must be pointed out that the lateral dynamic is reproduced with more accuracy by the model than the vertical dynamic. In the vertical direction, it has been observed that roll angular velocity, $w_{x}$, measured by both IMUs coincides with the simulation results but, nevertheless, the estimated vertical acceleration, $a_{z}$, is lower that the experimental measurements. This is due to the fact that the track centre line irregularities were measured every 50 mm. That means high frequency irregularities with wave lengths smaller than 100 mm cannot be simulated by the model. These short wave length irregularities normally lead to significant vertical accelerations on the vehicle. In addition, the gaps between consecutive rail sections that introduce impact forces in the vertical direction when the vehicles passes over them are not included in the measurement of the track measurement that is used as input for the simulation model.

The frequency analysis done also shows a good accordance between simulations and experiments in all cases. It must be pointed out that in the frequency analysis of the vertical dynamics, a peak at a frequency of 7.1 Hz is observed in the experimental signals $a_{z}$ and $\omega_{x}$ in both the wheelset and the bogie frame. This peak, not detected by the proposed model, is mainly related to a vibration mode associated with the pitch rotation and the longitudinal displacement of the different bodies. This explanation has been corroborated by the use of a full 3D coupled dynamic model which has been able to capture this vibration mode. In the lateral direction, an excellent fit is achieved between simulations and experimental measurements highlighting the comparison between the lateral acceleration, $a_{y}$, and yaw angular velocity, $w_{z}$, measured by both inertial sensors.

Looking forward, once the proposed model has been experimentally validated using the scaled railway and track, it can be then applied to the computational simulation of full scaled vehicles provided an equivalent model development methodology is followed. Another interesting point of the proposed simulation model is its high computational efficiency. In fact, the model implementation in Matlab is real time capable when dealing with ideal tracks without irregularities. It is expected that an implementation in C++ will allow the model to carry out real time simulations even considering irregular tracks.

## Figures and Tables

**Table 1:** Experimental scaled track ideal geometry.

Section	Length	Section Type
A	22 m	Tangent
B	3 m	Transition
C	26 m	Constant radius
D	3 m	Transition
E	6 m	Tangent
F	3 m	Transition
G	12 m	Constant radius
H	3 m	Transition
I	12 m	Tangent
